# Intergenerational effects of violence on women’s perinatal wellbeing and infant health outcomes: evidence from a birth cohort study in Central Vietnam

**DOI:** 10.1186/s12884-021-04097-6

**Published:** 2021-09-23

**Authors:** Huyen Phuc Do, Philip R. A. Baker, Thang Van Vo, Aja Murray, Linda Murray, Sara Valdebenito, Manuel Eisner, Bach Xuan Tran, Michael P. Dunne

**Affiliations:** 1 Queensland University of Technology (QUT), Faculty of Health, School of Public Health and Social Work, Brisbane, Australia; 2 Institute of Health Economics and Technology, Hanoi, Vietnam; 3 Institute for Community Health Research, Hue University of Medicine and Pharmacy, Hue University, Hue, Vietnam; 4 Faculty of Public Health, Hue University of Medicine and Pharmacy, Hue University, Hue, Vietnam; 5 Department of Psychology, University of Edinburgh, Edinburgh, UK; 6 College of Health Sciences, Massey University, Wellington, New Zealand; 7 Institute of Criminology, University of Cambridge, Cambridge, UK; 8 Institute for Preventive Medicine and Public Health, Hanoi Medical University, Hanoi, Vietnam; 9 Department of Health, Behavior and Society, Johns Hopkins Bloomberg School of Public Health, Baltimore, USA; 10 Australian Centre for Health Law Research, Queensland University of Technology (QUT), Brisbane, Australia

**Keywords:** Pregnant women, Prenatal intimate partner violence, Mediation pathway, Violent victimization, Childhood maltreatment

## Abstract

**Background:**

Girls exposed to violence have a high risk of being victimized as adults and are more likely than non-abused women to have children who are treated violently. This intergenerational transmission may be especially serious when women suffer violence during pregnancy and early motherhood, as it impairs maternal wellbeing and infant health and development. This study examined the intergenerational effects of being exposed to childhood maltreatment (CM) and prenatal intimate partner violence (p-IPV) on perinatal mental distress and birth outcomes in central Vietnam.

**Methods:**

A birth cohort study in Hue City, Vietnam was conducted with 150 women in the third trimester of pregnancy (Wave 1) and 3 months after childbirth (Wave 2). Using multivariable logistic regression models, augmented inverse-probability-weighted estimators and structural equation modelling (SEM), we analyzed a theoretical model by evaluating adjusted risk differences and pathways between CM, p-IPV and subsequent perinatal adversity and indicators of infant health problems.

**Results:**

One in two pregnant women experienced at least one form of CM (55.03%) and one in ten pregnant women experienced both CM and p-IPV (10.67%). Mothers who experienced p-IPV or witnessed IPV as a child were approximately twice as likely to experience poor mental health during pregnancy [ARR 1.94, 95% CI (1.20–3.15)]. Infants had a two-fold higher risk of adverse birth outcomes (low birth weight, preterm birth, admission to neonatal intensive care) [ARR 2.45 95% CI (1.42, 4.25)] if their mothers experienced any form of p-IPV, with greater risk if their mothers were exposed to both CM and p-IPV [ARR 3.45 95% CI (1.40, 8.53)]. Notably, significant pathways to p-IPV were found via adverse childhood experience (ACE) events (β = 0.13), neighborhood disorder (β = 0.14) and partner support (β = − 1.3).

**Conclusion:**

These results emphasize the detrimental and prolonged nature of the effect of violence during childhood and pregnancy. Exposure to childhood maltreatment and violence during pregnancy increases the risk of maternal mental health difficulties and adverse birth outcomes. Antenatal care systems need to be responsive to women’s previous experiences of violence and maternal mental health. The significant protective role of partner support and social support should also be considered when designing tailored interventions to address violence during pregnancy.

**Supplementary Information:**

The online version contains supplementary material available at 10.1186/s12884-021-04097-6.

## Background

Intimate-partner violence (IPV) is a pervasive public health, criminal justice, and social concern that affects nearly four in 10 women during their life-course [1, 2]. Pregnancy is one of the most critically sensitive periods when a mother’s history of victimization in childhood may heighten exposure to IPV in adulthood [3, 4]. The combination may have an inter-generational impact on her infant. Around 28% of pregnant women experience physical or sexual IPV during pregnancy [5] and a recent umbrella review of 12 studies found that emotional p-IPV can be wide ranging, with between 1.8 to 67.4% [6]. p-IPV is also associated with a range of adverse perinatal outcomes, such as miscarriages and pre-term births [7]. Extensive research has shown a strong relationship between p-IPV exposure and low maternal wellbeing (e.g. depression [8], suicidal risk [9], substance abuse [10]) and poor birth outcomes [11, 12].

The “intergenerational transmission of violence” or “cycle of violence” can refer to two processes. First, children who were exposed to violence personally or who witnessed IPV as children become tomorrow’s perpetrators of violence against their children or spouses in adulthood [13]. Second, this term also refers to the risk of being re-victimized in adulthood. Studies of risk factors suggest early exposure to violence is linked to a higher risk of involvement with violence in later life [14]. Women who were victims of sexual abuse as a child have a higher risk of being sexually assaulted in adulthood [15]. Childhood experiences can create ongoing interpersonal and social vulnerabilities for re-victimization via IPV in adulthood.

The intergenerational nature of violence is evident, as mothers who were maltreated as children are more likely to perform harsh punishment and intensive discipline and display hostility toward their child [16]. A child whose mother experienced IPV may have twice the risk of being exposed to psychological aggression and violent discipline (e.g. shouting or slapping) [17]. A recent study based on UNICEF’s Multiple Indicator Cluster Survey in 21 low and middle-income countries (LMICs), [18] has shown that women who experienced childhood corporal punishment are twice as likely as other mothers to have a positive attitude toward inter-partner aggression and violence being “acceptable” in certain situations. However, to date, the intergenerational transmission of p-IPV is not adequately understood. The overall prevalence of p-IPV in LMICs is approximately double the burden reported in high-income countries [5]. A recent review of 112 articles revealed that most studies examining the adverse effects of CM and p-IPV on child outcomes have primarily been implemented in high-income countries, with few in LMICs [19].

Given the interconnections between various forms of IPV, it is important to understand the developmental trajectories of violence over time, across the life course, and through generations, and document re-victimization from childhood to motherhood. Identifying the mediating effect of mothers’ experiences of violence as a child on their likelihood of experiencing p-IPV and the intergenerational effects of experiencing p-IPV on later birth outcomes could help highlight the points where the cycle of violence can be broken through early interventions. Murray et al. (2018) argued that p-IPV can significantly affect child outcomes via the intergenerational effects of maternal mental disorders, maternal-infant bonding, substance misuse, suboptimal nutrition, reduced utilization of prenatal health-care services, and infectious diseases during pregnancy [19]. A systematic review synthesizing 19 studies showed that women who experienced IPV were more likely to have low-birth-weight infants [OR 1.18 (95% CI 1.05–1.31)] and preterm birth [OR 1.42 (95% CI 1.21–1.63)] [11]. Although the pathways between p-IPV exposure and unplanned Cesarean labor [20] and complicated delivery [21] are well established, little has been done to investigate important links between violence from childhood to motherhood, and to consider whether such links predict associations with neonatal wellbeing.

A recent review of 15 primary studies revealed that p-IPV is a significant problem in Vietnam, affecting approximately 32% of pregnant women [22]. There is an important link between p-IPV and maternal mental disorder and birth outcomes [23] or infant social-emotional development [24] in the northern [25, 26] and southern areas of Vietnam [27]. However, to date, few studies in LMICs have investigated the buffering effect of neighborhood characteristics and interpersonal relationships (e.g., social support, partner support) on p-IPV severity, and intergenerational impacts on birth outcomes (e.g., delivery mode and neonatal conditions). Brown et al. (2020) suggested an important focus of research with birth cohorts should be to examine the impact of family or environmental factors and neighborhood disorder on early childhood development [28].

A growing body of literature reinforces the impact of neighborhood characteristics such as social support [29]; neighborhood connectedness [30]; and support of family, friends and partners on the level of IPV and women’s wellbeing. Existing research recognizes the critical role played by the buffering effect of informal social support (i.e. friends, family) on improving quality of life and individual wellness among victims of IPV [31, 32]. More recently, emerging literature has offered contradictory findings regarding the protective mechanisms of these factors and their mediating effects on IPV severity. Some studies have found that more social support might correlate with higher IPV episodes [33], as the perpetrators may use protective factors (e.g., social cohesion) to obscure coercive control behind closed doors [29].

Therefore, a richer understanding of the main, mediating effects of social and neighborhood connections in contemporary Asian contexts is important to optimize future anti-violence intervention. This study examined the effects of childhood maltreatment (CM) and p-IPV on perinatal wellbeing and birth outcomes. Multiple socio-ecological factors were considered as precursors and mediators. We hypothesized that women who experienced CM and/or p-IPV would be at higher risk of poor maternal mental health and adverse birth outcomes and be more likely to have positive attitudes towards corporal punishment for their child. This research aims to build evidence regarding the effect of violence trajectories to inform violence prevention interventions. The objective of this study was to measure recall of childhood violence experiences and recent IPV during pregnancy and examine intergenerational pathways to explain associations with perinatal mental distress and birth outcomes in Central Vietnam.

## Materials and methods

### Study design

This was a prospective birth cohort study of women conducted in Hue City, Vietnam. Mothers were recruited at the third trimester of pregnancy (Wave 1, W1) and re-approached 3 months after childbirth (Wave 2, W2). The cohort study in Vietnam was conducted as part of the “Evidence for Better Lives Study - EBLS”, co-led by a consortium of academics from the University of Cambridge, UK and eight middle- income countries. The research protocol has been reported elsewhere [34]. A stratified multi-stage cluster sample of pregnant women was recruited between May to October 2019. The aim was to recruit 150 pregnant women, all 18 years older, living in Hue City, and in the third trimester of pregnancy. One hundred and fifty pregnant women were recruited from 195 eligible participants, achieving a participation rate of 71.6% for the baseline survey (W1). Three months after childbirth, 148 mothers (98%) agreed to participate in the follow-up interview (W2). Two mothers whose newborns died in utero or after delivery were not re-approached for the follow-up interview. This study complied with the ethics requirements obtained by the QUT Human Research Ethics Committee in Australia (Approval Number: 1900000082), Hue University of Medicine and Pharmacy, Vietnam (Approval Number: H2018/430) and the University of Cambridge, UK (Approval Number: 18/180).

### Data collection and study measurements

The English questionnaire was forward translated into Vietnamese based on WHO guidelines [35]. A pre-pilot interview with five pregnant women was conducted to ensure the cultural adaptation of the Vietnamese version was acceptable to participants. Women participated in face-to-face interviews combined with both computer-aided personal interviews (CAPI) and audio-supported self-completion interviewing (A-CASI) for some sensitive questions (e.g., adverse childhood experiences). The fieldworker was available to provide technical assistance [36]. Validation of the survey instrument has been published elsewhere [37]. All participants were screened and provided written informed consent form before the interview.

#### Socio-demographic characteristics

Women reported their socio-demographic characteristics including age, occupation, ethnicity, mother’s educational attainment, family structure, and pregnancy history (e.g., number of pregnancies, number of antenatal check-ups). Additionally, the “MacArthur Scale of Subjective Social Status” was used to measure a sense of social status. This visualized ladder indicates a “social ladder where people stand in” from 0 to 10 points [38] and a higher score indicates a high level of perceived social status.

#### IPV victimization

Intimate partner violence during pregnancy was measured using a scale adapted from the WHO’s Multi-country Study on Women’s Health and Domestic Violence against Women [39]. This scale was a self-reported measure of both lifetime IPV and how often abuse had been experienced in the past 6 months, with responses on a 4-point Likert scale ranging between “never” = 1 and “many times” = 4. p-IPV frequency was estimated by a total score of 13 items as a continuous variable, ranging from 0 to 39. Higher scores represented worse experiences of p-IPV. Scores for three types of violence, including emotional abuse (four items), physical abuse (six items), and sexual abuse (three items) were summed from individual items. This scale has previously been validated among pregnant women in Vietnam [40]. The total score for three sub-scales in the present study shows good internal consistency reliability (alpha = .7 to .89).

#### Maternal wellbeing and birth outcomes

Maternal mental health was reported using combined mental health scales to measure wellbeing – WHO5 Perceived Stress Scale, Perceived Stress Scale 10 items (PSS10), and Patient Health Questionnaire – 9 items (PHQ9-depression scale). A dichotomous variable of poor mental health (Yes/No) was generated from any mental health difficulties (low wellbeing or high antenatal distress or moderate or severe depression). The psychometric testing for three scales has been reported elsewhere and suggested that the combined measures of stress, wellbeing, and depression can be more sensitive to classify pregnant women with health problem in Vietnamese population [37]. The Attitudes about Physical Punishment Scale, designed by Deater-Deckard et al. (2003), was used to measure endorsement of physical punishment by parents [41]. There are five items with a response on a 5-point Likert scale, ranging from “Strongly disagree = 1” to “Strongly agree = 5” Higher scores indicated greater endorsement of corporal punishment (Cronbach alpha = .85). Adverse birth outcomes were dichotomously generated (Yes/No) if the infant had low birth weight (LBW - weight less than 2500 g at birth) or preterm birth (PTB - born before 37 weeks of pregnancy) or being admitted to neonatal intensive care. These self-report variables were verified by the research team via data from the national program for maternal and newborn health at commune health center.

#### Predictors

Childhood abuse, witnessing IPV as a child, and experiencing significant family dysfunction during childhood were estimated from responses to 19 out of 31 items in the Adverse Childhood Experiences International Questionnaire (ACE-IQ) [42]. This is a retrospective measurement of ACE events that has been tested in international cross comparative studies including among Vietnamese students [43]. The domains included experiences of psychological, physical, and sexual abuse; living with household members who were abused or suffering mental illness, or misusing substances, at risk of suicide, having been in prison, being orphaned, parental divorce or separation; and/or experiencing childhood neglect. The ACE variable was generated from the total score combined form of CM (i.e. experienced two forms of CM, Yes/No), witnessing IPV as a child (Yes/No), parental separation/ divorce (Yes/No). This scale showed acceptable internal consistency (alpha = .72).

Perceived partner supportiveness captured mother-father relationship quality based on Goldberg and Carlson (2014) according to a 5-point Likert scale from “Never = 1” to “Always = 5” [44]. A total score was summed from individual five items, ranging from 5 to 25. Perceived social support was partly reflected via the adapted Multidimensional Scale (MSPSS) of Perceived Social Support (support that a woman believed would be forthcoming if needed). This tool was developed based on perceptions of support from three levels: family, friends, and a significant other [45]. Based on 7-points of the original instrument, we adapted the 5-point Likert scale, ranging from “Strongly disagree = 1” to “Strongly agree = 5”. The social support variable score was a total of 12 items and higher scores indicate better perceived social support, Cronbach’s alpha in this study was .75 for the family subscale, .95 for friend subscale, and .89 for support from significant others. Neighborhood disorder was measured by nine items about neighborhood/social disorder including litter in the streets, safety road, garbage, traffic, vandalism, street fights and drunken gangs on street [46]. Item responses were summed to produce a total score from nine items on a 4-point Likert scale from “Not a problem” = 1 to “A large problem” = 4 (Cronbach alpha = .81). Neighborhood cohesion was measured using scale extracted from the study of Mujahid (2007) [47]. The response was 4-Likert scale, ranging from “Strongly disagree =1” to “Strongly agree =4”. A greater score showed stronger neighborhood closure. Intergenerational closure was measured using a subscale developed by Sampson et al. (1999) [48]. The response was 4-point Likert scale, ranging from “Strongly disagree = 1” to “Strongly agree = 4”. A greater score showed stronger intergenerational closure. The total score was a continuous variable summed from four items (Cronbach alpha = .84). At the follow-up interview, the mothers were asked about their delivery mode “How was your baby delivered?”*,* and those who responded to “an unplanned, emergency caesarean section (C-section)”, “forceps delivery” or “vacuum delivery” were categorized as instrumental or surgical delivery (“Yes/No”).

### Theoretical model

**Fig. 1:**
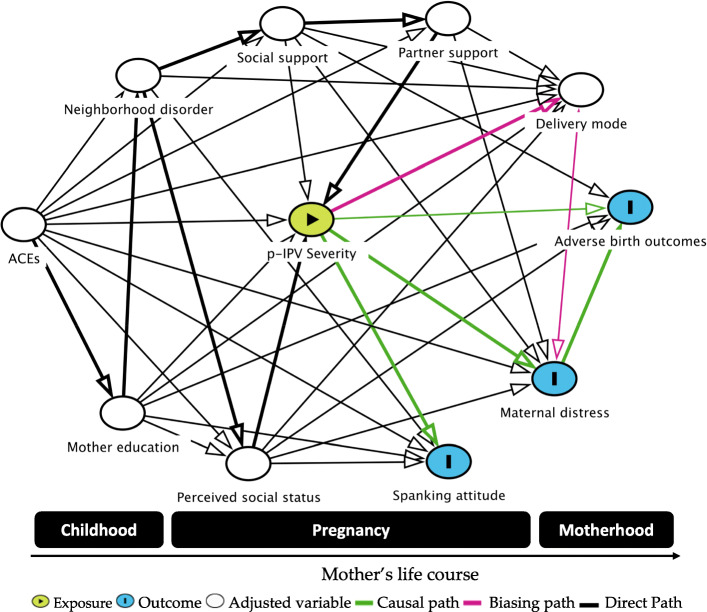
Theoretical model of pathways between violence exposure and birth outcomes

To investigate the association between violence victimization during childhood and pregnancy and birth outcomes, a theoretical model was designed using DAGitty, software for drawing and analyzing causal diagrams (Directed Acyclic Graphs – DAGs that was publicly accessed at http://www.dagitty.net/) [49]. This graphic parametric structural equation model **(**Fig. 1**)** represents three causal pathways (green lines) from p-IPV (exposure) to attitudes towards corporal punishment, prenatal distress, and adverse birth outcomes (outcomes). In addition, the principle of the algorithm [50] also established the minimum set of covariates or mediators necessary for adjustment and suggests potential biasing pathways or confounded associations (pink lines). The covariates in the graphical model established by the DAG for adjustment set [51] included ACE history, mother’s education, perceived social status, neighborhood characteristic, social supports, partner supports and delivery mode, preterm birth, maternal parity, smoking during pregnancy, mother’s schooling, and age.

### Statistical analysis

Initially, the extent of missing data was explored. All items showed a low level of missing data (from 0 to 0.2%). Further analysis of latent variables was performed to determine normal distribution via the skewness index (> 3) and kurtosis index (> 10) [52] (See Table 1–3, Supplementary Document for descriptive statistics of measures).

To adjust the effect of confounders and identify associations between p-IPV and maternal mental disorders, multivariable logistic regression with a stepwise backward approach was then carried out to examine the extent to which differing covariates contributed to generate the weights for use in the augmented-inverse-probability-weighted (AIPW) model. Four multivariable logistic regression models were tested for p-IPV (Yes/No), poor mental health during pregnancy (Yes/No), adverse birth outcomes (Yes/No), and prenatal mental health (Yes/No). The covariates included all theoretically relevant measured covariates at individual, inter-personal and structural levels of the socio-ecological model (e.g., demographic and pregnancy characteristics, partner/social support, neighborhood characteristics). The stepwise selection was processed by forward addition and backward removal of the interactions once no further significant interactions were entered. Following this, variables associated with this outcome in bivariate analysis with a *p* < 0.20 were evaluated for inclusion in the multivariate model manually in a forward stepwise manner (*p* < 0.1). Only statistically significant variables with the dependent variable were retained in the final model to reduce the probability of statistical power loss. Odds ratios (OR) and 95% CI were calculated for variables included in the final model.

Next, after fitting the logistic regression models with a set of observations, the adjusted risk ratios (ARR) and 95% confidence interval for the ARRs were computed based on *adjrr* – a Stata’s margins command – for the transform-the-endpoints method [53]. This transform-the-endpoints method resulted in an asymmetric confidence interval performs better for a dataset with small sample sizes [54]. The AIPW estimator used the *teffects ipw* command to compute the inverse-probability weights based on the parameters obtained from logistic regression models above [55]. To estimate adjusted risk differences (ARD) or average treatment effect, AIPW models were generated for p-IPV (treatment level) and three outcomes (adverse birth outcome, prenatal mental health, and corporal punishment attitude). The AIPW approach is considered a doubly robust method to address both incomplete data and model misspecification, using the exponential conditional mean model for outcome and the heteroskedastic probit for exposure (p-IPV) [56, 57].

Finally, structural equation modeling (SEM) using the bootstrapped maximum likelihood method was applied to obtain robust standard errors (SE) with 5000 replications, which is suggested to provide adequate power when sample size lower than 200 [58]. In this model of pathway analysis, p-IPV severity score, history of CM (Yes/No), antenatal stress score, emergency delivery (Yes/No), birth outcome (Yes/No), PSS10 (continuous variable), and theoretical related factors were performed to reveal the significantly correlated factors. The model fit was inspected based on various indices, including the root-mean-squared error of approximation – RMSEA (<.06), comparative fit index – CFI (>.9), standardized root mean squared residual-SRMR (<.08), Tucker-Lewis Index – TLI (>.9), and Chi-square/df indices [59]. Due to the sample size and the model’s complexity, maternal socio-demographic characteristics were not stratified in the models. The correlations were interpreted and compared using standardized path coefficients that are normally less affected by the different scales (i.e. variables were converted to z-scores before running analysis) [60]. All statistical tests were performed using STATA version 15.0 (Stata Corp. LP, College Station, TX, USA) and the significance level was set at *p* ≤ 0.05.

## Results

### Maternal and socio-demographic characteristics

Table 1 shows the demographic characteristics of the sample. Most women (71.62%) were employed (blue-collar or white-collar labor), the mean age was 29.86 years (range 19–47 years), and nearly half of the women had completed post-secondary education (trade school, technical college, or university). Approximately 13% of participants rated themselves as having low socioeconomic status compared to other families in Hue City (rating of 3 or less on a scale from 1as the lowest to 10 as the highest).

**Table 1:** Demographic and prenatal characteristics of pregnant women in Hue city (*n* = 150)

A. Demographic and prenatal characteristics	Total n (%)	B. Pattern of victimization	N (%)
**Mother age (years), mean [SD]**	29.86 (5.01)	**Mono victimization**	42 (28)
**Household wealth score, mean [SD]**	7.28 (1.41)	Lifetime IPV	24 (16)
**Occupation**		p-IPV victimization	22 (14.67)
Unemployed	42 (28.38)	At least one form of CM	82 (55.03)
Blue-collar workers	46 (31.08)	Childhood emotional abuse (CEA)	67 (44.67)
White-collar workers	60 (40.54)	Childhood physical abuse (CPA)	50 (33.33)
**Highest educational attainment**		Childhood sexual abuse (CSA)	9 (6)
Up to primary school	28 (18.92)	Childhood neglect	2 (1.33)
Secondary school	29 (19.59)	Witnessing violence	110 (73.33)
High school	23 (15.54)	CM and p-IPV	16 (10.67)
Vocational training, university or higher	68 (45.95)	**Combination of two forms**	40 (26.67)
**Low social status** (Yes vs. No)	19 (12.84)	IPV and witnessing	19 (12.67)
**Prior pregnancy risk** (Yes vs. No)	69 (46.6)	CPA and witnessing violence	46 (30.67)
**Current pregnancy illness** (Yes vs. No)	27 (18.2)	CSA and witnessing violence	9 (6)
**Adequate antenatal service used** (Yes vs. No)	12 (14.67)	CEA and witnessing violence	61 (40.67)
**Poor prenatal mental health** (Yes vs. No)	27 (18)	CPA and IPV	15 (10)
**Support of spanking attitude** (Yes vs. No)	112 (74.67)	CSA and IPV	2 (1.33)
**Delivery mode**		CES and IPV	16 (10.67)
Vaginal	76 (51.35)	CPA and CSA/neglect	2 (1.33)
Planned caesarean	41 (27.7)	CPA and CEA	35 (23.33)
Instrumental caesarean	31 (20.95)	CEA and CSA/neglect	8 (5.33)
**Neonatal care admission (Yes vs. No)**	11 (7.43)	**Combination of three forms**	
**Adverse birth outcomes** (Yes vs. No)	38 (35.33)	CEA, IPV, and witnessing violence	31 (20.67)
**Baby sick (Yes vs. No)**	22 (14.86)	CPA, IPV, and witnessing violence	13 98.67)
**Exclusive breastfeeding failure** (Yes vs. No)	59 (39.86)	CSA, IPV, and witnessing violence	2 (1.33)
**Postpartum depression (PPD)**		CEA, CPA, and witnessing violence	8 (5.33)
No symptoms	110 (74.32)	CPA, CPA, and witnessing violence	34 (22.67)
Mild depression	32 (21.62)	CSA, CPA, and witnessing violence	5 (3.33)
Moderate or severe depression	6 (4.05)	CPA, CSA, p-IPV, witnessing violence	1 (0.67)

*Note*: *CEA* Childhood emotional abuse, *CSA* Childhood sexual abuse, *CPA* Childhood physical abuse

Two-thirds of the participants were multiparous (71.6%), and almost half reported experience difficult pregnancies before the current pregnancy (e.g., miscarriage, abortion, preterm birth, stillbirth, or severe persistent vomiting). One-fifth of the women reported some current pregnancy-related illness such as gestational diabetes, heart disease, or anemia, and 6.1% reported a problem had been detected with the fetus, such as oligohydramnios and growth restriction. Approximately 18% reported moderate to high levels of symptoms of mental health problems (i.e. distress, lower wellbeing, or depression). Only 15% of the women reported that they had fully utilized antenatal services including four or more visits for vaccination for rubella and tetanus, iron supplementations, ultrasound screening, and psychological counselling. Notably, four in five women supported using corporal punishment as a form of child discipline.

One-fifth of the women experienced instrumental/surgery assisted delivery. Approximately 10% of newborns required special care nursery and 15% of infants experienced illnesses (e.g., vomiting, diarrhea, or low weight problems). One in three women had infants who experienced LBW, PTB or neonatal intensive care admission, with 15% of infants having an illness by 3 months postpartum (e.g. vomiting, diarrhea, weight disorder). Approximately one in three women had a sign of mild to severe postpartum depressive symptoms, and 40% of mothers were unable to exclusively breastfeed their baby after 3 months.

**Fig. 2:**
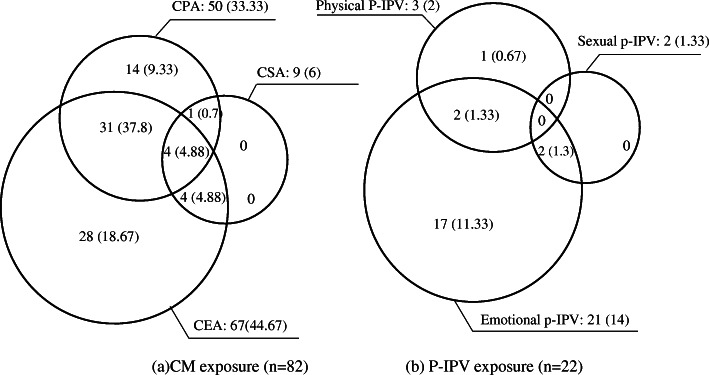
The pattern of victimization of violence during childhood and motherhood

Figure 2 shows a pattern of multiple victimizations during childhood and motherhood. More than half of the women reported exposure to one or more type of child maltreatment, primarily emotional abuse (44.67%) followed by physical violence (33.33%), and 26.67% of women experienced combined forms of CM. Approximately 15% of women considered themselves to be victims of p-IPV, primarily due to emotional violence (14%). One in 10 women (10.67%) experienced both CM and p-IPV.

Table 2 shows the factors associated with p-IPV experience, maternal mental health during pregnancy, attitudes about physical punishment, and adverse birth outcomes after controlling for other variables (demographic characteristics and covariates retained in the multivariable logistic regression model). Experience of childhood maltreatment, father’s education, mother’s education, living in a local community with a low level of neighborhood closure, and a low level of prenatal health service utilization was strongly associated with p-IPV victimization. Notably, the ARR estimate on prenatal mental health showed that women who experienced p-IPV or witnessed IPV as a child were two times more likely to have poor mental health during pregnancy than were non-abused women. Women whose husbands had low formal education were 2.4-times more likely to experience poor mental health during pregnancy [ARR = 2.79 95% CI (1.16–6.71, 95% CI]. The infants had a two-fold higher risk of adverse birth outcomes (LBW, PTB, admission in neonatal intensive care) [ARR = 2.45 95% CI (1.42, 4.25)] if their mothers experienced any form of p-IPV. The risk was substantially higher if the mothers were exposed to both CM and p-IPV [ARR = 3.45 95% CI (1.40, 8.53)]. Women with a history of parental separation/divorce and who witnessed IPV as a child were 90% more likely to have adverse birth outcome (ARR = 1.9), holding all else constant. The women’s attitudes about corporal punishment were significantly associated with childhood traumatic events [ARR = 1.07 95% CI (0.99–1.15)] and maternal education [ARR = 1.72 95%CI (0.96–3.10)].

**Table 2:** Multiple Logistic regression model and Augmented Inverse-probability- weighted (AIPW) model for ARR and ARD of p-IPV on mothers’ perinatal wellbeing and infant health outcomes

**A. Multiple Logistic regression model**	**Model 1: p-IPV victimization** **ARR (95% CI)**	**Model 2: Prenatal mental health ARR (95% CI)**	**Model 3: Attitude about spanking** **ARR (95% CI)**	**Model 4: Adverse birth outcomeARR (95% CI)**
**Individual factors**				
Mother’s education (secondary vs. higher)	0.78 (0.41–1.46)	1.61 (0.82–3.19)	1.72 (0.96–3.10)**	
Father’s education (secondary vs. higher)	6.9 (1.63–29.20)**	2.79 (1.16–6.71)**		1.41 (0.99, 1.99)*
Lack of prenatal care (Yes vs. No)	3.35 (1.80–6.23)**			
Combined forms of CM (Yes vs. No)	6.39 (1.69–24.23)***			
Unplanned pregnancy			1.32 (0.86–1.99)	
ACE score			1.07 (0.99–1.15)*	
**Interpersonal factors** (Yes vs. No)				
Family disruption		1.471 (1.04–2.481)		2.08 (1.19, 3.62)*
IPV witness as a child		2.13 (1.03–4.40)*		1.95 (0.95, 4.04)
At least one form of p-IPV		1.94 (1.20–3.15)*		2.45 (1.42, 4.25)*
Combined forms of IPV and CM				3.45 (1.40, 8.53)**
**Structural factors** (Yes vs. No)				
Lack of intergenerational closure	0.46 (0.23–0.89)*		1.27 (0.96–1.68)	
Lack of neighborhood cohesion			1.23 (0.96–1.56)	
**Fit indices**				
Pseudo R2/ VIF	0.30/ 1.14	0.16/ 1.15	0.13/ 1.07	0.12/ 1.25
Hosmer-Lemeshow chi2(8)	4.95	5.79	7.03 / 1.2	4.34
AIC/BIC	96.67/114.66	166.93/ 184.91	157.14/ 175.12	160.65/ 174.45
McFadden’s Adj R2/ Cragg & Uhler’s R2	0.20/ 0.39	0.25/ 0.09	0.06/ 0.199	0.05/ 0.19
**B. Augmented Inverse-probability- weighted (IPW) model**	**Model 1: Risk of CM on p-IPV victimization** **(Yes vs. No)**	**Model 2: Rik of p-IPV on prenatal mental health** **(Yes vs. No)**	**Model 3: Risk of p-IPV on attitude about spanking** **(Yes vs. No)**	**Model 4: Risk of p-IPV on adverse birth outcome** **(Yes vs. No)**
**Adjusted risk difference (95%CI)**	0.18 (0.09–0.27)***	0.40 (0.16–0.64)***	0.18 (−0.09–0.44)	0.77 (−2.60–4.15)

Note: * p < 0.05; ** p < 0.01; *** p < 0.001

The AIPW models show the absolute risk measures (or ARD). The data can be interpreted as showing that women who experienced CM were much more likely than non-exposed women to also be exposed to p-IPV. The difference in risk was estimated at 18 percentage points. Women exposed to p-IPV in the previous 6 months had more mental health problems during pregnancy (higher by 40 percentage points). The differences for those who supported physical punishment (18 percentage points) and those who experienced adverse birth outcomes (77 percentage points) were also more substantial.

**Fig. 3:**
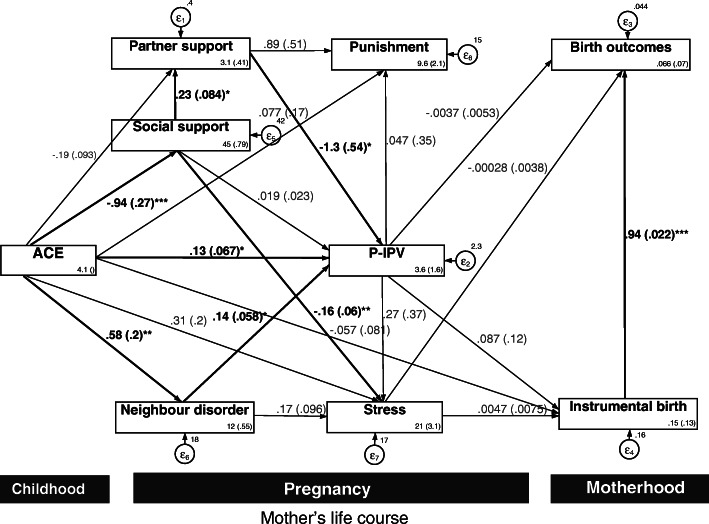
Mediation pathway of P-IPV, delivery method, birth outcome and associated factor with standard path coefficients. Model fit well (χ2 (15, n = 148) = 16.84 (p = 0.33), RMSEA (90% CI) = .0037 (.001; .09), CFI = 0.99, TLI = 0.97, SRMR = .0039. * *p* < 0.05; ** *p* < 0.01; *** *p* < 0.001

Figure 3 illustrates the path model for longitudinal data with bootstrapped SEs (based on 5000 replications) to test associations between p-IPV and related birth outcomes. This model explained 20.74% of the overall variance and 30.33 for p-IPV severity. The path model showed acceptable fit as the indices were in the acceptable range [χ2 (15, *n* = 148) = 16.84 (*p* = 0.33), RMSEA (90% CI) = .0037 (.001; .09), CFI = 0.99, TLI = 0.97, SRMR = .0039], with no modification indices below 3.4. The significant pathways are indicated by bold paths. Three out of four observed variables showed significant total effects to p-IPV, including ACE (β = 0.13), neighborhood disorder (β = 0.14) and partner support (β = − 1.3). No significant pathway was found between p-IPV, antenatal distress and attitude toward physical punishment of children and birth outcomes. However, a significant direct pathway was found between instrumental birth and birth outcomes in this model (β = 0.94) [See Table 4, Supplementary Document for further indirect testing].

## Discussion

This study contributes several useful insights. First, to our knowledge, this is one of the first studies to show evidence of intergenerational effects of child maltreatment and violence during pregnancy on mothers’ mental distress and the health of their infants in Vietnam. Evidence from our birth cohort extends the fairly limited international research into violent trajectories from childhood to pregnancy; clearly, these phenomena share many risk factors [61]. Second, this study shows the risk differences and pathway effects of p-IPV severity upon maternal wellbeing and infant health outcomes. The longitudinal data allowed us to investigate causal relationships via various robust models (i.e. AIPW estimators) to address the missing data issue. Importantly, findings from this study reinforce the long-term consequences of child abuse throughout the lifespan and its impact on maternal wellbeing.

Our study suggests the pattern of p-IPV in Central Vietnam (14% of emotional IPV and 3.3% of physical and sexual IPV) is similar to the global prevalence (13.8%–physical P-IPV, 8%–sexual P-IPV, and 28.4%–emotional P-IPV) [5]. A recent national study examining violence against women in Vietnam found a similar prevalence of physical p-IPV (3.4%) [62]. However, this prevalence was lower than the findings of studies with Vietnamese mothers in our recent review (30% for emotional p-IPV and 5% for physical IPV) [22] and lower than the prevalence among women in a survey in northern Vietnam (35.3%) [26] and among female patients seeking fertility treatment in a southern city (16.8% for emotional violence, 7.3% for physical violence, and 12.4% for sexual violence) [63]. This difference might reflect the significant influence of Buddhist values of kindness, belief in karma, non-violence and Nirvana (the state of peace and happiness, achieved after giving up three poisons of greed, hatred, and delusion), which are especially strong in the contemporary population of Hue City [64]. Future studies with randomized sampling throughout the diverse nation of Vietnam to further understand which contextual factors significantly contribute to the apparently lower prevalence of p-IPV in the central region.

At the individual level, a clear implication to emerge from the analysis is the cumulative and prolonged harmful effects of CM, p-IPV victimization, witnessing parental IPV, and growing up amid family dysfunctions. The effects were apparent for adverse birth outcomes and mental health difficulties during pregnancy. Women who reported a history of CM were more likely to experienced p-IPV. The detrimental effect of p-IPV and childhood trauma then doubled the risk of poor maternal mental health and adverse birth outcomes. This is consistent with other research in South Africa [65]. The intergenerational effect of victimization is also consistent with studies that show women who have experienced multiple adverse events have more stress and trauma-related symptoms in the last trimester than women with low exposure [4] and a dose-response relationship was found between multiple incidents of abuse and the highest levels of psychological distress [66]. Particularly, a recent survey found that women who suffered severe IPV had a 4.5-fold increased likelihood of LBW and PTB infants, compared to those with low-level IPV exposure. p-IPV is strongly associated with antepartum hemorrhage, which puts the unborn baby at increased risk of PTB, LBW and long-term health complications in early childhood [67].

The findings at the interpersonal level, while preliminary, suggest that greater partner support during pregnancy can help to reduce the onset of p-IPV. Our findings broadly support the work of other studies that link partner connectedness with maternal wellness [68, 69], pregnancy outcomes [70], and IPV exposure [21]. Partner support may have a buffering effect to improve maternal wellbeing and birth outcome via promoting mothers’ personal feeling of security, relationship stability and marital satisfaction [71]. A low level of partner support can be an important indicator of inadequate caring, listening and understanding that contributes to emotional neglect and abuse [72].

At structural levels, we found the important predictors of p-IPV included neighborhood disorder and non-partner social support. Exposure to neighborhood disorder could contribute to a higher risk of both p-IPV and mental distress. This result aligns with Beyer’s (2015) findings that higher perceived neighborhood disorder is associated with increased IPV [73], especially “street” crime [74] or neighborhood disorganization (e.g., drug trading, shootings). Both IPV and CM are high in areas with neighborhood disorders and residential instability [75]. Based on a review of 36 studies, Voith (2017) suggested that the effect of neighborhood disadvantage might interact with the individual’s subjective disorder to contribute to IPV perpetration [30]. A 24-year longitudinal study also suggested that people perceived more positive neighborhoods as less likely places to experience IPV and individual experiences of violence are reinforced by the observing of neighborhood risk factors (e.g. alcohol or drug misuse, neighborhood violence observed) [76]. Negative views of human behaviors and conflict relationships can be fostered during pregnancy by a perception of threatening and dangerous environments that turn to feelings of isolation and powerlessness and trigger physiological responses (e.g. anxiety, anger, fear and depression) [77]. Our findings underscore the importance of contextual factors related to neighborhood disadvantages and how women can be protected against violence by improving neighborhood safety in future intervention toward ending violence. Future work focusing on a series of protective and promotive neighborhood factors could potentially reduce the trajectory of p-IPV, build resilience for survivors of IPV, and promote a harmonic family and optimal child development [28].

Perceived social support is a potential protective factor for p-IPV, as it fully mediated the effects of the history of ACE and perceived partner support during pregnancy. Our analysis found that women who perceived a high level of social support had less antenatal distress and a lower risk of p-IPV. This finding aligns with previous studies that suggest poor social support magnifies the harm from IPV [78]. Low family support may double the risk of repeated IPV episodes [79]. Notably, social support was strongly associated with partner support via possible interference of influential family members in the patriarchal system. The positive impact of social support suggests that enhancing social support might be beneficial to improving maternal mental health and reducing the burden of ACE exposure and further investigation should focus on how family, friends and social disadvantage may influence children’s development via violent intimate relationships [28].

This paper contributes to debates concerning the inter-generational transmission of IPV in relation to social attitudes that tolerate child corporal punishment. Violence experienced by mothers may lead to harsh parental educational practice. A previous study suggested that children whose mothers experienced IPV have double the risk of being exposed to psychological aggression and using violent methods (e.g. shouting or slapping) to discipline their children [17]. Similar results were found in UNICEF’s Multiple Indicator Cluster Survey in 21 LMICs, [18], which showed that women who experienced childhood corporal punishment are twice as likely to have a positive attitude toward IPV acceptability. Longitudinal data have also suggested dose-response between higher level of IPV exposure with higher degrees of spanking parental behavior [80]. This current study revealed that four in five women (74.67%) endorsed spanking children. However, this study did not find a significant risk difference between attitude to child spanking and p-IPV victimization. This unexpected finding can be explained by the small number of women who experienced p-IPV (22 participants) and the pervasive proportion of women supporting child spanking (75%). Thus, our sampling power may not have been able to detect a small difference. This hypothesis is supported by the fact that corporal punishment is prevalent among Vietnamese parents, as three in four children aged 1–14 experience corporal punishment or violent discipline [81]. Further, corporal punishment is more common during school age in Vietnam [82]. “Tiger parenting” practice is a common violent parental discipline toward high academic performance for children, and is especially more frequently in urban high-income families in Vietnam [83]. Hence, a further study could assess the long-term effects of p-IPV on perception toward corporal punishment and pathways to physical punishment behaviors using a larger sample size.

One of our unexpected findings was the negative direct effect of physical child abuse history on instrumental/surgery delivery (e.g. C-section or forceps or vacuum). This is a controversial topic with mixed evidence. A systematic review of 43 studies found minimal effects of childhood abuse experienced on a high level of instrumental/surgery delivery (i.e. forceps, vacuum extraction or cesarean section [84], while a recent study found that women who were sexually or physically abused in childhood had a higher level of fear of childbirth and were more likely to experience a complicated vaginal birth. Specifically, women raped in childhood had a considerable greater risk for C-section (OR 15.7, 95% CI 5.0–49.1) or assisted vaginal birth (OR 13.1, 95% CI 4.9–34.5) [85] or more difficult pregnancies and delivery [86]. It is difficult to explain this unexpected result; however, it may be related to the high proportion of planned C-section among abused women [87], perhaps due to worry about their infant’s health [88]. In the Vietnamese cultural context and contemporary central Vietnam, some women may believe that a child may be born dead as punishment or karma [89].

There are limitations to this study due to reliance on subjective self-reported data and possible recall bias especially for childhood experience events that may have occurred one to three decades earlier. However, the self-administered questionnaire via tablet to assist confidentiality may have encouraged participants to disclose their adverse experiences more openly than in face-to-face interviews [90]. Additionally, the sample size is a significant limitation for the external validity of this study. However, we believe that the bootstrapping technique and AIPW modeling may have helped to estimate robust SE values and avoid type I error in testing risk differences. Further, although convenience sampling could limit generalizability, the participants were recruited from eight out of 27 commune health centers in Hue City; thus, the sample was diverse and may be similar to urban settings in central Vietnam. Finally, this study was unable to examine some other forms of violence that could be experienced during a woman’s life span (e.g. bullying, dating violence) that may be harmful to pregnant women. Future birth cohort studies should include more comprehensive measurement of traumatic events.

## Conclusions

This study highlights the inter-generational effects of p-IPV on maternal mental disorders during pregnancy and adverse birth outcomes. We found important pathways relating to support from partners, social support, and neighborhood disorder that affected p-IPV experiences and subsequent perinatal distress among Vietnamese women. Tailored interventions should focus on promoting women’s social support services and encourage the engagement of partners in gender equality programs.

## Supplementary Information

**Additional file 1: Table S1**. Mean score of measures. **Table S2**. Descriptive statistics for indicators of latent variables (*n* = 148). **Table S3**. Pearson correlation matrix among measured variables. **Table S4**: Mediation analysis for indirect effect based on Monte Carlo test.

## Data Availability

The datasets used and/or analysed during the current study are available from the corresponding author on reasonable request (Huyen P. Do - phuchuyen@gmail.com).

## Abbreviations

AIPW: Augmented-inverse-probability-weighted
A-CASI: Audio-supported self-completion interview
CAPI: Computer-aided personal interviews
CFI: Comparative fit index
CHC: Commune Health Center
CM: Childhood Maltreatment
EBLS: Evidence for Better Lives Study
LMICs: Low and middle-income countries
LBW: Low birthweight
PTB: Preterm birth
PDD: Postpartum depression
PHQ9: Patient Health Questionnaire – 9 items
p-IPV: Prenatal intimate partner violence
PSS10: Perceived Stress Scale
RMSEA: Root-mean-square error of approximation
RMSR: Root-mean-square residual
SEM: Structural Equation Modelling
SRMSR: Standardized-root-mean-square residual
WHO5: WHO Well-Being Index
