# Supplementary material for: Intergenerational effects of violence on women’s perinatal wellbeing and infant health outcomes: evidence from a birth cohort study in Central Vietnam
Source: BMC Pregnancy Childbirth. 2021 Sep 23;21:648. doi: 10.1186/s12884-021-04097-6 (PMC8461881; doi:10.1186/s12884-021-04097-6)
Supplement: Supplementary file 1 — Additional file 1: Table S1. Mean score of measures. Table S2. Descriptive statistics for indicators of latent variables (n = 148). Table S3. Pearson correlation matrix among measured variables. Table S4: Mediation analysis for indirect effect based on Monte Carlo test. [file 12884_2021_4097_MOESM1_ESM.pdf]

Table S1. Mean score of measures.

Variable	Items	Scale Range	Mean	SD	Min	Max
History of physical & sexual child abuse (ACE)	19	0-19	2.51	2.03	0	10
P-IPV severity	13	0-39	0.51	1.84	0	16
Perceived social status score	1	1-10	5.09	1.39	1	9
Neighbourhood disorder score	9	9-36	31.37	4.39	17	36
Intergenerational closure score	4	5-20	7.77	2.57	4	16
Density of friendship ties	2	2-10	5.04	2.16	2	10
Perceived stress scale	10	10-40	17.52	4.52	10	29
Wealth index	13	0-13	7.28	1.41	3	10
Partner supportiveness	5	5-25	19.49	3.35	7	25
Number of antenatal check-up	1	1-40	6.82	2.36	2	17
Social support	12	12-60	25.24	7.04	12	51
Support from health staff	5	0-100	77.67	14.2	40	100
Attitude to corporal punishment	5	5-25	13.3	3.9	5	22

Table S2: Descriptive statistics for indicators of latent variables (n = 148)

Scales	No of items	Mean (SD)	Median (Range)	Skewness	Kurtosis	Cronbach's alpha
Emotional P-IPV	4	1.1 (0.37)	1 (1-3.8)	4.8	25.9	0.83
Physical P-IPV	6	1 (0.07)	1 (1-1.83)	10.9	125.5	0.89
Sexual P-IPV	3	1 (0.06)	1 (1-1.7)	9.6	98.7	0.7
Total score	13	0.5 (1.8)	0 (0-16)	5.5	39.4	0.81
PHQ-9 after childbirth	9	3.2 (2.8)	3 (0-12	0.96	3.7	0.79
MSPSS– Family	4	4.2 (0.5)	4.25 (1.75-5)	-0.7	5	0.75
MSPSS – Friend	4	3.5 (0.9)	4 (1-5)	-1.1	3.5	0.95
MSPSS – Significant other	4	3.9 (0.7)	4 (1-5)	-1.1	4.6	0.89
ACE-IQ- Adapted	19	2.5 (2.0)	2 (0-10)	0.8	3.5	0.72
Neighbourhood cohesion	5	1.8 (0.6)	1.6 (1-3.6)	0.8	3.4	0.84
ACE-IQ- Adapted	19	2.5 (2.0)	2 (0-10)	0.8	3.5	0.72
APP	5	3.3 (0.78)	3.4 (1.6-5)	-0.3	2.5	0.85
Neighbourhood/social disorder	9	1.5 (0.5)	1.3 (1-3.1)	1.1	3.6	0.81
Partner Supportiveness	5	3.9 (0.7)	4 (1.4-5)	-0.9	4.5	0.79
INSIRE recovery scale	5	77.6 (14.2)	80 (40-100)	-0.7	4.2	0.81

Note: MSPSS: The Multidimensional Scale of Perceived Social Support, P-IPV: Prenatal intimate partner violence, PPD: Postpartum depression, ACE-IQ: Adverse Childhood Experiences International Questionnaire, APP: Attitudes about physical punishment

Table S3: Pearson correlation matrix among measured variables

Variable	1	2	3	4	5	6	7	8	9	10
1. P-IPV severity	1									
2. Neighborhood closure	-0.13	1								
3. Neighborhood safety	-0.19	-0.26	1							
4. Social status	-0.12	-0.03	0.02	1						
5. Wealth index	-0.18	0.01	0.07	0.44	1					
6. Partner support	-0.50	0.00	0.02	0.25	0.22	1				
7. Social support	0.11	0.25	-0.04	-0.29	-0.09	-0.28	1			
8. Antenatal distress	0.20	0.23	-0.23	-0.10	-0.14	-0.37	0.28	1		
9. History of child abuse	0.29	0.05	-0.27	-0.22	-0.08	-0.25	0.28	0.28	1	
10. Antenatal check-up	-0.09	-0.01	0.01	0.18	0.20	0.20	-0.24	-0.17	-0.16	1

Table S4: Mediation analysis for indirect effect based on Monte Carlo test

Pathway	B (SE)	95% CI	IE	DE	RIT	RID
1. **ACE → Social support → Partner support**	**-0.021 (0.011)***	-0.045, -0.004	0.021	0.062	0.257	0.347
2. **ACE → Social support → P-IPV**	-0.029 (0.017)	-0.069, -0.002	0.029	0.019	2.852	1.54
3. **ACE → Partner support → P-IPV**	0.081 (0.057)	-0.004, 0.214	0.080	0.133	0.377	0.606
4. **ACE → Neighborhood disorder → P-IPV**	0.034 (0.023)	-0.002, 0.087	0.035	0.133	0.207	0.261
5. **ACE → P-IPV severity → Antenatal stress**	0.036 (0.059)	-0.070, 0.171	0.036	0.314	0.103	0.115
6. **ACE → P-IPV severity → Attitude toward physical punishment**	0.006 (0.052)	-0.101, 0.117	0.006	0.077	0.075	0.081
7. **ACE → P-IPV → Instrumental/surgery delivery**	0.003 (0.005)	-0.006, 0.013	0.003	0.011	0.290	0.225
8. **Social support → Partner support → P-IPV**	-0.029 (0.017)	-0.069, -0.002	0.029	0.019	2.852	1.540
9. **Neighborhood disorder → P-IPV → Antenatal stress**	0.016 (0.027)	-0.032, 0.078	0.016	0.167	0.089	0.098
10. **P-IPV → Emergency delivery →Baby sick**	0.002 (0.005)	-0.006, 0.014	0.002	0.014	0.151	0.178

Note: (*) * $p < 0.05$; ** $p < 0.01$; *** $p < 0.001$ based on Monte Carlo for indirect effect testing, IE: indirect effect, DE: direct effect, RIT: Indirect effect / Total effect, RID: Indirect effect / Direct effect.

Table S4 demonstrates the mediation analysis for the indirect effect of path analysis. Although ACE events exerted direct effects on partner support, the Monte Carlo test confirms the significant indirect effect on decrease partner support via perceived social support (β = -0.021). This means that approx. 26% of the effect of ACE on partner support is mediated by social support. No significant indirect effect was found in other pathways.
